# A Qualitative Analysis to Understand Perception about Medication-Related Problems among Older Minority Adults in a Historically Black Community

**DOI:** 10.3390/pharmacy10010014

**Published:** 2022-01-07

**Authors:** Ivy O. Poon, Felicia Skelton, Lena R. Bean, Dominique Guinn, Terica Jemerson, Ngozi D. Mbue, Creaque V. Charles, Uche A. Ndefo

**Affiliations:** 1 Department of Pharmacy Practice, Texas Southern University, Houston, TX 77004, USA; creaque.charles@tsu.edu (C.V.C.); Uche.ndefo@tsu.edu (U.A.N.); 2 Center for Innovations in Quality, Effectiveness and Safety, Michael E. DeBakey VA Medical Center, Houston, TX 77030, USA; felicia.skelton2@va.gov; 3 H. Ben Taub Department of Physical Medicine and Rehabilitation, Baylor College of Medicine, Houston, TX 77030, USA; 4 Aging and Intergenerational Resources, Division of Student Services, Texas Southern University, Houston, TX 77004, USA; lena.bean@tsu.edu (L.R.B.); terica.jemerson@tsu.edu (T.J.); 5 Department of Health Kinesiology and Sports Studies, Texas Southern University, Houston, TX 77004, USA; dominique.guinn@tsu.edu; 6 Nelda C. Stark College of Nursing, Texas Woman University, Houston, TX 77030, USA; nmbue@twu.edu

**Keywords:** medication-related problems, older adults, minority group, polypharmacy, multiple chronic illnesses

## Abstract

Older adults taking multiple chronic medications experience an increased risk of adverse drug events and other medication-related problems (MRP). Most current literature on medication management involves researcher-driven intervention, yet few studies investigate patients’ understanding of MRP in a diverse community setting. This report investigates patients’ perception of MRP and patient-centered strategies among a cohort of the older adult group in a historically Black urban community. The study design is qualitative using structured open-ended questions in a multidisciplinary patient-centered focus group. Patients (age 65 years or older) taking seven or more medications were recruited. The group comprises patients, caregivers, pharmacists, health educators, a physician, and a nurse. Recordings of the group discussion are transcribed verbatim and analyzed using thematic content analysis and categorized by codes developed from the social-ecological model. The group reports patient-provider relationships, previous experience, fear of side effects played important roles in medication adherence. There is an unmet need for medication management education and tools to organize complex medication lists from multiple providers. This study provides important insights into MRP experienced by minority older adults and provided researchers with potential strategies for future interventions.

## 1. Introduction

Medication-related problem (MRP) is a prevalent and critical health problem in the older adult population [1]. MRP encompasses any drug-related event that interferes or potentially interferes with optimizing a patient’s outcome [2]. Common examples of MRP include adverse drug events (ADE), therapeutic competition, medication non-adherence, and inappropriate drug use in older adults [2]. In a retrospective review of 1000 hospitalized older adults conducted by Kanann et al. [3], 18.7% experienced at least one ADE within 45 days after a hospital discharge, and approximately 35% of the ADE identified were preventable, and 37% resulted in severe or life-threatening outcomes [3]. A national surveillance analysis of 58 emergency departments (ED) across the US has found that individuals 65 years and older accounted for 34.5% of ED visits for ADR, and 43.6% of those resulted in hospitalization [4]. Lorganpai, S.J. has found that about 22.6% of older adults in the US have received a drug regimen with therapeutic competition, receiving at least one drug that may worsen a coexisting condition [5].

Multimorbidity [6], polypharmacy [7,8], physiological changes with aging [9] may increase the risk of MRP. African Americans (AA) are at higher risk of MRP due to the higher prevalence of chronic illnesses such as cardiovascular disease, hypertension, diabetes, dementia, stroke, cancer, and multimorbidity than all other racial and ethnic groups [10,11,12]. AA residing in historically Black medically underserved urban communities may experience additional challenges in medication literacy, limited access to quality pharmacy services, lack of transportation, lower socioeconomic status, and higher cumulative race-related stressors [13,14]. A cross-sectional analysis of Medicare data reported that AA patients were 21–34% less likely to be eligible for medication therapy management (MTM, Washington, DC, USA) services, a pharmacy service paid by Medicare for a comprehensive medication review, due to a higher generic dispensing ratio and using a medication discount plan due to low socioeconomic status [15]

Prior research studies on MRP in the AA population have shown that polypharmacy, multimorbidity, multiple providers, and inappropriate medication use are high and negatively impact humanistic and economic outcomes [16,17,18,19]. However, most studies have been quantitative and researcher-driven and have less emphasis on patient engagement in research design. As a result, there is a lack of knowledge about what MRP means for AA older adults with multiple chronic illnesses, perceived barriers to medication management, and appropriate strategies that meet their needs. This research study sought to fill this gap in knowledge by exploring the understanding of MRP perceived barriers and strategies in medication management in a diverse minority older adult group with polypharmacy. 

In 2018, the authors of this article created a patient engagement workgroup in a historically Black community, Third Ward, Houston, funded by the Patient-Centered Outcome Research Initiatives (PCORI, Washington, DC, USA). The long-term goal of this initiative is to increase health equity for the minority (historically Black) communities in medication and chronic disease self-management by engaging the targeted population in all stages of the research process. The workgroup consisted of multidisciplinary research investigators representing medicine, nursing, pharmacy, health education, and social work to engage patients in identifying research problems [20]. Patients recruited from the community with no prior relationships with the investigators were treated as equal partners in research, following the PCORI engagement rubric instead of study participants [21]. Patients had the right to vote to modify research strategies (patients’ votes have double weight) and made decisions following a strategic plan developed by the workgroup. The investigators presented key literature on strategies to improve medication management adapted to patients’ literacy and led discussions about MRP. The objectives of this study were to conduct a qualitative analysis of the workgroup meetings to (1) understand what MRP meant for this cohort, (2) potential barriers, and (3) strategies to improve medication management.

## 2. Methods

### 2.1. Study Design and Settings

This study’s approach was grounded theory using inductive reasoning and interpretive paradigm. Patients were engaged using the Patient-Centered Outcomes Research Institute (PCORI, Washington, DC, USA) engagement rubric for research [21]. The research protocol was reviewed and approved by Texas Southern University’s IRB board before the conduct of the study. The study was carried in the Third Ward community, located southeast of Houston downtown, with 14,295 residents, where 67% of the residents are non-Hispanic Black, and 51% had annual income under USD 25,000 [22]. In addition, it is a historically Black community and home to the fourth-largest Historically Black College in the US [23], Texas Southern University, Houston, TX, USA. A previous publication has reported details about study design, recruitment, and patient survey [20].

### 2.2. Patient Recruitment

This study’s principal investigator (IP) had multiple previous collaborative experiences with the Director of the Center on the Family, who established relationships and served as a gatekeeper to the community members. The principal investigator and the Director hosted two community outreach events to introduce the investigators, develop trust, and initiate medication management dialogue before patient recruitment. Patients were recruited as consultants, not study participants. Patients were recruited through the University’s Center on the Family senior group. Inclusion criteria were older adults aged ≥65 years old and self-reported taking ≥ seven chronic medications. 

Patients participated as part of a workgroup consisting of other members, including caregivers, a physician, a nurse, two pharmacists, a researcher/pharmacist, and three health educators. The rationale for including patients into the multidisciplinary researcher workgroup was to engage patients as partners in research based on the patient-centered outcome research principles [21]. The health professionals are research investigators providing insights on their practice experiences and sharing evidence-based literature to guide discussions about medication-related problems that are meaningful to the community members (patients).

### 2.3. Activities

The principal investigator (IP) led the discussions at the workgroup meetings. After two meetings, there were no new themes generated by the discussion. Therefore, the data collection has reached a saturation point. The investigator led an additional meeting to confirm no new themes [24]. The meetings were held in person after the older adult exercise group meetings. Two meetings were held in the meeting rooms of a community center, and one was held in a meeting room in the University’s recreation center. 

The workgroup spent three one-hour sessions to discuss a set of structured open-ended questions adapted from the asset-based community development (ABCD, Chicago, IL, USA) process principles to collect feedback on problems related to multimorbidity and polypharmacy and potential strategies to overcome the problems [25]. The questions asked were: (1) What community resources have been most helpful for seniors taking multiple medications to improve medication safety? What helps you to be better informed about how to take your medications? (2) How can the Houston community better support seniors taking multiple medications based on existing strengths? What are needs not being met? (3) What help would you need the most right now to improve medication safety?

### 2.4. Data Collection and Analysis

Recordings of the meeting were transcribed verbatim by two trained pharmacy students and cross-checked for omissions. Qualitative data analysis was performed by two independent professors and two pharmacy students on a research rotation. Each investigator read the transcripts individually to identify the common themes. Codes were developed based on the social-ecological model. The socio-ecological model describes the interactive characteristics of multiple levels (intrapersonal, interpersonal, community, institution, policy) that affect patient behavior and outcomes [26]. Each investigator (IP, FS) coded the transcripts according to the coding framework in Table 1. The investigators cross-checked and discussed the coding assignment for discrepancies. The qualitative analysis was conducted in Altas ti, Inc, Berlin [27]. 

## 3. Results

There were three workgroup meetings comprised of 12, 11 and 13 attendees, respectively. Table 2 shows the workgroup’s composition. The health professional members were the same individuals in the three meetings (physician, nurse, caregiver, pharmacists, health educators, social worker). All attendees were African Americans, except the principal investigator of the project was Asian. The meetings were held at the Emancipation Park and the University’s Recreation Center after the patient participants’ routine group activities. 

### 3.1. MRP Identified by the Workgroup

#### 3.1.1. Themes

**Figure 1:**
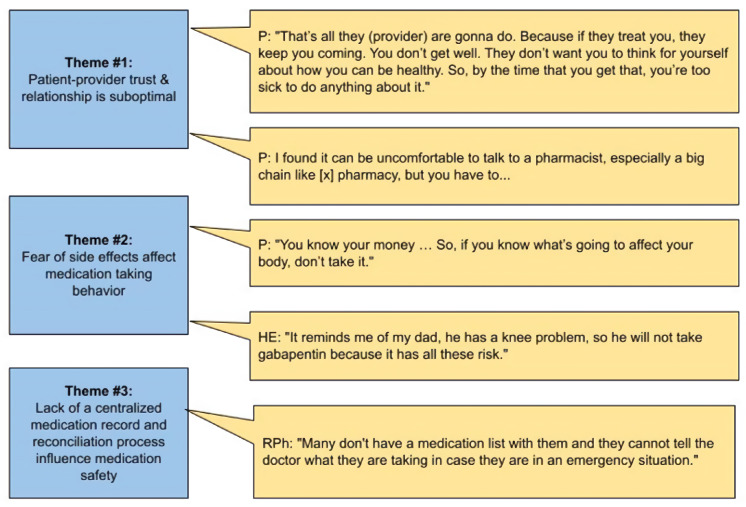
MRP identified by workgroup members. P = patients, HE = health educator, RPh = pharmacist.

The investigators identified three main themes related to MRP among workgroup participants (Figure 1). First, patients stated that trusting relationships with their doctors strongly influenced their medication adherence. Patients valued providers’ welcoming gestures and communication skills that showed genuine care. Second, workgroup members shared many stories about how previous experiences or messages from self, friends, and family affected medication adherence. For example, the fear of becoming addicted to pain medications after hearing from different media outlets (prescription patient information and the opioid crisis in the news) prevented some patients from filling their prescriptions despite doctors’ recommendations. Third, many participants with polypharmacy did not have and maintain an updated medication list, despite medication reconciliation being conducted in the clinics and hospitals. The relationships among the themes were analyzed in a network map and found in this article’s Supplementary Material Section.

#### 3.1.2. MRP by Social Ecological Model

The MRP identified by the workgroup was tagged and organized by the social-ecological model to stratified problems in different levels [26] (Table 3). 

When discussing how community pharmacy can help, a patient expressed that speaking up to ask a pharmacist can be intimidating (Figure 1). Several patient participants described the medication counseling as brief and pertained only to the medication dispensed. One said, “Normally if it’s a new prescription, there is a consultation right there. But a lot of times, you are just talking about one medication, without the others.” Another patient said, “the conversation (by the pharmacist) is usually one or two sentences. Is it for this? How do you take it? And if you have any questions?” One patient was very concerned about drug-drug interaction. She said, “The problem I have is if you’re taking three or four different medications, I’m always concerned about how each medication has its own set of problems. Because if you already have problems because otherwise, you wouldn’t be taking medications. Then the medication creates 5 or 6 other problems. And they give you 3 medications, and that’s another 10 problems. And you got 15 additional problems added to what’s wrong with you. So, it’s not … on you getting well at all.”

The patient participants disliked the drug information sheet that was given from the pharmacy. They said the drug information was too overwhelming, discouraged them from taking medicine. One patient said, “That makes me don’t want to take it (the medication).” A second patient said, “And if you read all these side effects, I stop reading them. I keep them for 30 days, and then if nothing happens, I will shred them. I keep it, and I won’t read it. If something starts happening, I will go back and read it. I stopped reading it.” A third patient said she would, “listen to what the doctor says because it’s going to be a shorter list than what the pharmacy says… because the pharmacy is always going to try to cover everybody, so that is going to give you all the possibilities, so the list is going to be really long.” A fourth patient described a similar experience, and she said, “It is on the sheet that the pharmacist gives you. And I was having the same problem. I was having a reaction, but I didn’t realize it was the medication. So, I had to dig and dig and dig and find that little sheet that is attached to that bottle, and I thought I had food poisoning.”

### 3.2. Potential Strategies Identified by the Workgroup

Strategies identified by the Workgroup are found in Table 4. The workgroup discussed whether using high-level technology was acceptable to seniors. Several patients showed excitement about using technology, saying, “that will be really neat,” “it’s also great for emergency personnel when they ask what you are taking,” and “that sounds wonderful.” However, they mentioned several barriers to implementing high-level technology such as a smartphone application (app), including “not everyone has access to a smartphone.” They suggested that an alternate approach, such as the wallet card, would be helpful for seniors who did not have smartphones.

A patient described her experience with getting information through messaging the physician, and she said, “They have that app …I took some tests yesterday, and the chart showed me what the doctor’s results and everything. And at the bottom is said “Ask a question,” and it said I had arthritis. And I had taken several x-rays and tests, so I had to send her a question saying, “Arthritis, where?” In my back or my knees. In a minute, she responded right back.”

## 4. Discussion

This study provides a patient-centered qualitative approach to understand MRP experienced by a cohort of older AA adults with polypharmacy and multiple chronic illnesses in a historically Black urban community. The results found that fear of side effects and a lack of a comprehensive medication list were the key problems experienced by this population group. In addition, trust towards providers was a strong mediator to getting the prescription filled and taking it as prescribed. These findings are similar to previous studies in that shared-decision making, and trust with provider mediate medication adherence in AA patients [28,29], and fear of side effects negatively impact medication adherence [30,31]. 

The workgroup in this study suggested an unmet need for skills and tools to create and maintain a comprehensive medications list by the patient to communicate with providers at different health systems using electronic charting systems that do not synchronize records. Additionally, the medication list maintained by each health system and pharmacy may not be completed if a patient receives care from multiple health systems and takes over-the-counter medications. 

Although all patient participants had polypharmacy, none were aware of MTM services provided by pharmacists. The Medicare set cost-based eligibility criteria to pay pharmacist-led MTM services, including a comprehensive medication review and list, for older adults with multiple medications [32]. Previous studies have shown that AA are less likely to receive MTM service than whites due to discount medication plans and generic medications [33]. Patient workgroup members emphasized that it is important to empower patients with the training and tools to create a medication list in an electronic spreadsheet or paper, medication wallet cards, or an app if a smartphone was available. 

Overall, patients were open to using high technology strategies, including using a computer or an app using a smartphone. It was mentioned in the workgroup that not all the older adults in their community had access to or had the skills to utilize an app on a smartphone. This concern resonates with a previous study reporting barriers in navigating medication organization app among older adults [34]. Several patients experienced communicating with their providers via messaging and found it helpful. 

One strength of this study is the established relationships with the patient participants and the investigators before the study. The trust helps to facilitate an open dialogue to discuss problems and strategies during each workgroup meeting. 

There are multiple limitations to this study. Due to the exploratory nature of this study, the patient sample size is small. The small number of participants in each meeting allows a round table discussion, but there is a potential the result may not represent the population. After the workgroup meetings, a community town hall survey (*n* = 69) was conducted to overcome this limitation. In [20], sixty-one (61)% of participants agreed that fear of ADEs and drug interactions from multiple providers was a problem in the survey. Sixty-five (65)% reported a lack of a personal comprehensive medication list. 

The presence of health professionals and caregiver in the workgroup may introduce bias to the patients’ responses. However, none of the patients had a previous patient-provider relationship with the health professional workgroup members, and their roles were patient consultants instead of participants. All workgroup members, regardless of occupations and categories, were treated equally as research partners. Patients’ opinions were highly valued in this PCORI project, in which patients’ votes counted double in each decision-making process of the workgroup. Another limitation is that all workgroup members were female except one male health educator. Further studies will be needed to explore potential extrapolation to the older male population. Finally, some absences among workgroup members in the three meetings may affect the findings.

## 5. Conclusions

This study highlights the need for strategies to reduce medication-related problems for the minority and underserved. The patients’ knowledge about medication and self-management skills are modifiable and may improve medication self-efficacy and adherence. Future studies will be needed to understand the social determinants of health and multi-level influence on medication self-efficacy and develop a community-driven medication self-management training program for the underserved and AA minority older adults. 

## Figures and Tables

**Table 1:** Codes used for qualitative analysis.

Problems	Research Strategies
Patient level	Patient level (low technology and high technology)
Intrapersonal level	Intrapersonal level
Community level	Community level
Health care system level	Health care system level
Policy level (cost and insurance)	Policy level

**Table 2:** Workgroup Composition.

Attendees Categories	First Meeting	Second Meeting	Third Meeting
Patients, *n*	2	3	3
Caregiver, *n*	1	1	1
Physician, *n*	1	1	1
Nurse Practitioner, *n*	1	1	1
Pharmacist, *n*	3	2	3 (2 ^1^ RPh, 1 ^2^ FQHC pharmacy director)
Health Educator, *n*	3	3	3
Social Worker, *n*	1	0	1
Total Number of Attendees	12	11	13

^1^ RPh = registered pharmacist, ^2^ FQHC = federally qualified healthcare center.

**Table 3:** Problems caused by multimorbidity and polypharmacy in respective levels.

Domains	Theme	Excerpt
Patient-level	The complexity of medication regimen, forgetfulness, and lack of organization	*P ^1^: “I normally take my meds in the morning…I have a routine, but I just forget.”*
	Experience adverse drug events	*P ^1^: “I didn’t sleep at all last night. I cramped so much because it took much water off of me. It hurt. Going to the restroom all night, trying to get cramps out of my foot and toes.”*
	Lack of trust in provider’s recommendation	*P ^1^: “…difficult to take what my doctor prescribed because it may not do me good.”*
Intrapersonal and community-level	Medication sharing	*P ^1^: “And it’s common for them to knock on their neighbor’s door and say hey you know that blood pressure pill, can I have a pill?”*
Health system level	Excessive prescribing, polypharmacy, and confusion	*RPh ^2^: “The X health system’s EMR (electronic medical record) is not linked to other facilities. So, if that patient goes to Y or Z hospitals, the X hospital doesn’t know what they are taking over there... All we know is solely based on patient’s report. And if they don’t have a print out of what they received, we will be going by the patient’s word.”* *MD ^3^: “You do the best you can when they are in your office. But you know that it’s not enough.”* *P ^1^: “I had many specialists. One gave me one medication, and the other gave me one medication. And if I have gone to the hospital, I still have all these medications. You’re taking these medications because they told you to take them. It’s horrible because instead of starting with 3 medications, you had about 7–8 medications that everybody is telling you to take.”*
Policy level	Insurance drug coverage drug cost	*P ^1^: “It’s very common for them to want to hoard, especially with things that they know are very expensive. I think it’s very common for them to give themselves less or have …. to make sure it stretches.”*

^1^ Quotations from different patients, ^2^ from a pharmacist, ^3^ from a physician.

**Table 4:** Strategies by social-ecological levels.

Domains	Theme	Excerpt
Patient-level	Low-tech: Being able to communicate with health care providers was the most important strategy to improve medication management	*P ^1^: “We have to learn how to communicate with them so they can help you. Don’t be afraid to ask.”*
	Low-tech: Medication organization tools that were helpful included pillboxes, wallet cards with medication listed, placemat with the medication schedule	*P ^1^:”I like the idea about the wallet and what I would suggest is to keep an index card in your wallet. When crisis happened, they can pull out the index card with allergies and drugs.”*
	High-tech: Create a medication list using Excel, use an app	*P ^1^:” “I think <investigator’s name> should address the issue, it sounds like we’re becoming so technical with technology coming into play. We used to say we’ll send you someone to help you deal with that. But not everyone has to know how to do these little things right here… So, we may be right on the curb of changing over into more electronic things for older adults to now do because it’s a whole different generation as you can see that is technically savvy. Maybe that is where we are heading in terms of multiple medications.”*
Intrapersonal level	Getting help from family members/friends who are health professionals	*P ^1^: “I am so grateful to be in this organization (project) because I like to helping elders because some of them they don’t really know. God is here for us for a purpose. We’re here to help one another and I thank God for the doctor.”*
Community level	Health fairs at churches and community centers	*P ^1^: “more ways to help you get rid of (properly dispose of) medication properly so it won’t get in the wrong hands or kids won’t touch it.”* *P ^1^: “attend a computer class to learn how to look up credible drug information.”*
Health system level	Medication reviews by a health care professional, receive a medication list, messaging with providers and see lab results through an app	*P ^1^: “it would be so helpful if when a doctor calls in your prescription, he can also call in all of the meds you use, so that the pharmacist can see where you get all of your medicines from and all the meds that you take.”* *P ^1^:”print out all of the medications they are receiving from that pharmacy?”*
Policy level	Classes funded by local health agencies, free cell phones/hearing aids offered by a state program for hearing impaired	*HE ^2^: “There are classes on chronic disease self-management, fall prevention, balance program offered by the City of Houston Area Agency on Aging. There was a HomeMed program that provided medication review through the City of Houston.”*

^1^ Quotations from different patients, ^2^ from health educator.

## Data Availability

The data presented in this study are available on request from the corresponding author. The data are not publicly available due to sharing of data was not in the original research protocol.

## Supplementary Materials

The following supporting information can be downloaded at: https://www.mdpi.com/article/10.3390/pharmacy10010014/s1. Figure S1: Qualitative themes analysis displayed in a network map.
