# Supplementary material for: A Qualitative Analysis to Understand Perception about Medication-Related Problems among Older Minority Adults in a Historically Black Community
Source: Pharmacy (Basel). 2022 Jan 7;10(1):14. doi: 10.3390/pharmacy10010014 (PMC8788468; doi:10.3390/pharmacy10010014)
Supplement: Supplementary file 1 [file pharmacy-1488172-supplementary.pdf › pharmacy-1488172-supplementary.pdf]

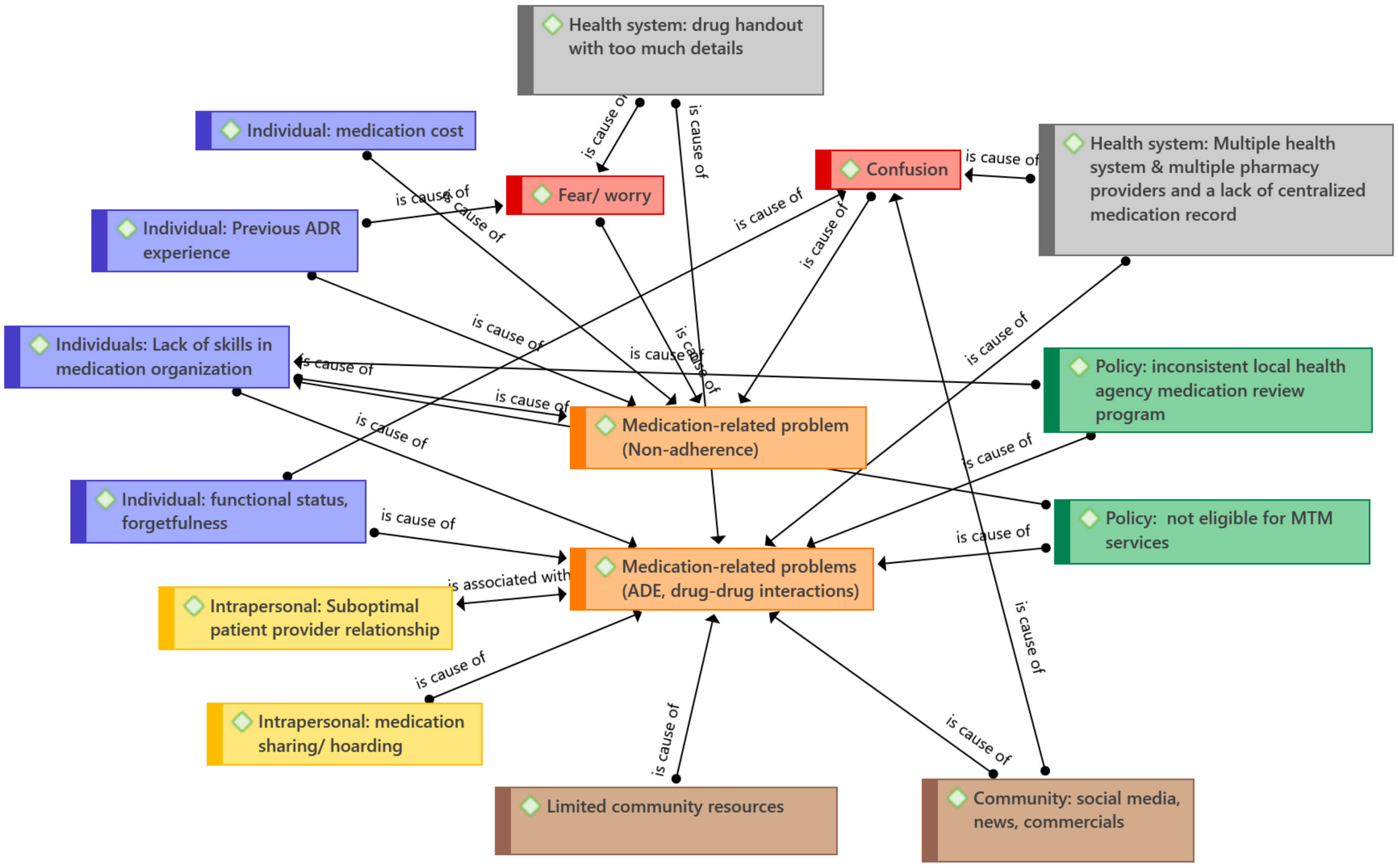

Figure S1. Qualitative themes analysis displayed in a network map.
